# Paget's disease of the skull causing hyperprolactinemia and erectile dysfunction: a case report

**DOI:** 10.1186/1752-1947-2-234

**Published:** 2008-07-18

**Authors:** Rachel Hepherd, Paul E Jennings

**Affiliations:** 1 ST2 Core Medical Training, Hull Royal Infirmary, Anlaby Road, Hull, HU3 2JZ, UK; 2 York Hospital, Wigginton Road, York, YO31 8HE, UK

## Abstract

**Introduction:**

Hyperprolactinemia is an uncommon cause of erectile dysfunction in men. Paget's disease of the skull is a relatively common disease. This case proposes a rare example of a causative link between the two and how treatment of the Paget's disease with bisphosphonates helped the patient regain erectile function.

**Case presentation:**

A 67-year-old man with Paget's disease of the skull presented with prostatitis, erectile dysfunction, and hyperprolactinemia. Radio-isotope scanning showed increased vascularity around the sphenoid bone. Treatment with intravenous bisphosphonates improved the active Paget's disease as indicated by declining alkaline phosphatase levels and the patient's erectile function while serum prolactin levels became normal and serum testosterone levels remained unchanged.

**Conclusion:**

It is possible that hyperprolactinemia is unrecognised in other patients with Paget's disease of the skull. Normalizing elevated prolactin levels by using bisphosphonates in treating Paget's disease appears to be more appropriate than traditional treatment for hyperprolactinemia.

## Introduction

We describe the case of a man who presented with erectile dysfunction secondary to hyperprolactinemia, an uncommon cause of erectile dysfunction in men. In addition, we discuss how the hyperprolactinemia arose, due to Paget's disease of the skull, which caused increased vascularity around the sphenoid bone, as proven by radio-isotope scanning.

## Case presentation

A 67-year-old man presented with recurrent renal calculi and prostatitis. During the course of the consultation, he also complained of erectile dysfunction. Subsequent biochemical investigations revealed a raised level of prolactin and he was referred to the endocrinology department.

The patient had a long-standing history of Paget's disease. Apart from erectile dysfunction, he had no lack of libido, gynaecomastia or galactorrhoea. He was not on any regular medication, and took only paracetamol as required. The full series of initial biochemical investigations is shown in Table 1. Hyperprolactinaemia was confirmed on serial resting samples (range 594 to 819 mU/litre) and repeat pre-10am testosterone levels remained in the low-normal range (16.40, 10.77 and 15.02 nmol/litre respectively). A computed tomography scan of the head was initially performed. This showed extensive Paget's disease extending into the sphenoid surrounding the pituitary gland.

**Table 1:** Initial biochemical investigations

	At presentation	After 24 months bisphosphonate	Normal ranges
Calcium	2.43	2.35	2.10 to 2.60 mmol/litre
Alkaline phosphatase	684	83	30 to 110 IU/litre
Follicle stimulating hormone	3.9	6.2	1.0 to 7.0 IU/litre
Luteinising hormone	4.2	3.7	1.0 to 8.0 IU/litre
Testosterone	20.20	14.50	10.0 to 31.0 nmol/litre
Prolactin	752	492	33 to 585 mU/litre
Thyroid stimulating hormone	1.75	1.97	0.1 to 5.0 mU/litre
Free T4	12.7	13.3	10 to 24 mmol/litre

The pituitary gland appeared normal on sagittal and coronal reconstructions therefore, the patient underwent a radio-isotope bone scan. This revealed increased bone activity within the skull vault, with further areas in the left petrous temporal bone and extending into the skull base in the region of the pituitary fossa.

The hypothesis was that the hyperprolactinaemia may have been consequent upon the increased vascularity and blood flow through the sphenoid secondary to the activity of the Paget's disease. The patient was treated with intravenous pamidronate (30 mg per week over 6 weeks) and both alkaline phosphatase and prolactin levels became normal, while the patient regained erectile function.

## Discussion

Erectile dysfunction is a common disorder and is an important health-care issue as it acts as a surrogate marker of other diseases of more concern, such as diabetes mellitus and peripheral vascular disease. The prevalence of erectile dysfunction increases with advancing age, and this is in part due to declining levels of Luteinizing Hormone (LH) and Follicle Stimulating Hormone (FSH), so-called hypogonadotrophic hypogonadism [1,2]. Although there may often be a psychological basis, organic causes must be considered and excluded (Table 2) [3].

**Table 2:** Causes and risk factors for erectile dysfunction [1]

Environmental	Exogenous	Metabolic	Neurological	Vascular	Others
Ageing	Smoking	Diabetes mellitus	Multiple sclerosis	Coronary and/or peripheral vascular disease	Radial prostatectomy
	Drugs	Hyperlipidaemia	Spinal cord injury	Aorto-iliac surgery	Radial cysto-prostatectomy
	Alcohol		Other neurological disorders	Hypertension	Blunt perineal and/or pelvic trauma
	Hormone treatments				Hyperprolactinaemia
					Psychogenic

Hyperprolactinemia is a relatively uncommon cause of erectile dysfunction in men and is most often caused by a microprolactinoma of the pituitary gland. Symptoms may also include diminished libido, infertility and more rarely, reduction in facial and body hair, galactorrhoea and gynaecomastia [4]. Prolactin inhibits the release of LH and FSH, directly impairing testosterone production [5]. Treatment of hyperprolactinemia/prolactinoma is via suppression of prolactin, either with medical therapy using dopamine agonists (eg bromocriptine or cabergoline) or transsphenoidal surgery [4,5]. Prolactin secretion is normally inhibited by dopamine flux down the pituitary stalk from the hypothalamus. Raised prolactin levels occur due to either a prolactin-secreting adenoma within the pituitary or from the lack of dopamine-mediated suppression from stalk dysfunction, which can occur if the stalk is distorted by intra- or extra-sella lesions, as we believe was the case in this patient. In this case, conventional treatment for hyperprolactinaemia would only be symptomatic, given that the underlying cause was not due to a prolactin-secreting tumour, but to the increased vascularity of the bones and associated deformity surrounding the pituitary gland, along with stalk dysfunction.

The response to treatment of hyperprolactinemia/prolactinoma is via measurement of normalizing prolactin, along with subjective improvement of symptoms, such as return of libido or erectile function [5].

Paget's disease of bone (osteitis deformans) is a chronic inflammatory remodelling disease first described by Sir James Paget in 1877 [6]. Any bone may be affected but most commonly it is the axial skeleton (spine, pelvis, femur, sacrum and skull) that is affected. The symptoms of Paget's disease include bone pain, skeletal deformity, pathological fractures and high-output congestive cardiac failure (increased vascularity and blood flow). Symptoms and complications in the skull include headache, skull enlargement (for example, frontal bossing) and deafness owing to compression of the eighth cranial nerve. More rarely, compression of the second, fifth and seventh cranial nerves can occur, if the disease directly disrupts the paths of these nerves [7,8].

The mainstay of treatment for Paget's disease is to control disease activity with bisphosphonates. This is the first reported case of Paget's disease affecting pituitary gland function by involvement of the sphenoid bone. In this case, a course of intravenous pamidronate was used successfully but other oral bisphosphonates can be used depending on disease severity [7,8].

## Conclusion

Hyperprolactinemia is an extremely rare consequence of a common bone disorder. Although there are other complications of Paget's disease related to increased vascularity of bone, we have not identified any such similar cases of pituitary hormone dysfunction in the literature. This complication was important to recognise as hyperprolactinemia was treated by treating the Paget's disease with bisphosphonates, and not with traditional therapies used for hyperprolactinemia/microprolactinoma.

## Competing interests

The authors declare that they have no competing interests.

## Consent

Written informed consent was obtained from the patient for publication of this case report and any accompanying images. A copy of the written consent is available for review by the Editor-in-Chief of this journal.

## Authors' contributions

PEJ was the physician responsible for the care of the patient. RH reviewed the relevant literature. Both RH and PEJ wrote the paper and edited the final manuscript prior to submission.
